# A study on the relationship between psychological resilience, vagal tone, and social adaptation: A non-pharmacological treatment perspective based on left-behind children

**DOI:** 10.1097/MD.0000000000045235

**Published:** 2025-11-07

**Authors:** Xuejing Liu, Longhuan Zhang, Meiying Zhang, Ningning Li

**Affiliations:** a Department of Psychiatry and Psychology, Harrison International Peace Hospital, Hengshui, Hebei, China.

**Keywords:** heart rate variability, left-behind children, mediation effect, psychological resilience, social adaptation, vagal tone

## Abstract

This study aims to explore the relationship between psychological resilience, vagal tone (heart rate variability [HRV]), and social adaptation, with a focus on the mediating role of HRV in the impact of psychological resilience on social adaptation. It also seeks to provide theoretical support for mental health interventions for left-behind children from a non-pharmacological perspective. This study employed a cross-sectional design with cluster sampling to select 2 middle schools in the urban-rural fringe of Zaoqiang County, Hebei Province, with a total of 312 left-behind children as the research subjects. Of these, 156 children were in the high-resilience group and 156 were in the low resilience group. The Youth Resilience Scale and the Adolescent Social Adaptation Scale were used to assess resilience and social adaptation. HRV was measured using portable equipment to assess high-frequency power (HF) and pNN50. Data analysis included descriptive statistics, analysis of covariance (ANCOVA), Pearson correlation analysis, mediation effect testing, logistic regression, and multiple linear regression. The high-resilience group showed significantly better HRV (HF and pNN50) and social adaptation compared to the low resilience group (all *P* <.001). Psychological resilience was significantly positively correlated with HRV (*R* = 0.59–0.81) and social adaptation (*R* = 0.95). Mediation analysis revealed that both HRV-HF and HRV-pNN50 partially mediated the relationship between psychological resilience and social adaptation (indirect effects = 0.25, 95% CI did not include 0). Logistic and multiple regression analyses further confirmed that psychological resilience (B = 2.12, *P* <.001) and HRV-pNN50 (B = 0.14, *P* = .005) were important positive predictors of social adaptation. Psychological resilience is significantly associated with better social adaptation among left-behind children, and this relationship may be partially explained by vagal tone (HRV), which plays a potential mediating role. This study provides physiological support for HRV-based non-pharmacological psychological interventions and holds significant theoretical and practical value.

## 1. Introduction

In recent years, with the rapid advance of urbanization and population mobility, a large group of “left-behind children” has emerged in rural China. According to data from the Ministry of Civil Affairs and the Ministry of Education, tens of millions of children live for extended periods without their parents, cared for instead by grandparents or other relatives.^[1]^ Deprived of parental care and emotional connection, these children face numerous challenges in emotional regulation, behavioral norms, interpersonal interactions, and social integration, and they show a heightened risk of psychological vulnerability during development.^[2]^ Importantly, the phenomenon of prolonged parent–child separation is not unique to China. Similar challenges have been documented among migrant families in Latin America, where children are frequently left behind due to parental labor migration (Nobles, 2011), and in European contexts where transnational migration has been linked to children’s emotional and social difficulties (Dreby, 2010). Viewing the issue within this broader global context underscores the international relevance of studying left-behind children.^[3]^

A substantial body of research has shown that left-behind children commonly experience difficulties in social adaptation, reflected in poor academic adjustment, strained peer and teacher relationships, and elevated risks of psychological problems. Social adaptation – defined as the ability to cope with environmental stress, fulfill social roles, and maintain mental and physical health – is a vital capacity for children and adolescents to navigate critical developmental periods and foster positive personality traits.^[4]^ Identifying the key factors that shape social adaptation and developing scientifically grounded intervention pathways has therefore become a central concern in psychology and sociology.

Psychological resilience is widely recognized as a core psychological resource that enables individuals to maintain functioning, recover quickly, and achieve positive development in the face of adversity.^[5,6]^ As a capacity for coping with stress and challenges, resilience not only buffers the impact of adverse environments but also supports emotional stability and goal orientation when children face loss, failure, or emotional deprivation, thereby promoting social adaptation.^[7]^ Studies consistently show that adolescents with higher resilience adapt more successfully to complex social environments and are at lower risk for psychological disorders. Internationally, resilience has also been conceptualized as a universal developmental asset. Fergus and Zimmerman (2005)^[8]^ proposed a resilience framework for youth health promotion, while Masten (2014)^[9]^ characterized resilience as “ordinary magic” that underpins positive adaptation across cultures. Nevertheless, examining the resilience–adaptation link solely from a psychological perspective is insufficient. Increasingly, researchers are turning to underlying physiological mechanisms, particularly the role of autonomic nervous system regulation.

Vagal tone, reflecting parasympathetic nervous system activity, is an important physiological indicator of self-regulation capacity and is commonly indexed by high-frequency power (HF) and pNN50 within heart rate variability (HRV) measures.^[9,10]^ HRV not only captures variability in cardiac rhythms but also serves as a window into brain–heart interactions, closely associated with emotional regulation, social behavior, and stress recovery.^[11]^ Higher HRV is generally associated with better emotional control, greater environmental sensitivity, and stronger psychological resilience. Thus, HRV may mediate the influence of psychological resilience on social adaptation – resilience enhances vagal tone, which in turn fosters more adaptive social outcomes.^[12]^ While this mechanism has been supported in some adult studies, systematic evidence in adolescents remains scarce, particularly in high-risk populations such as left-behind children.

At the same time, psychological interventions have shifted from relying primarily on pharmacological treatments and one-on-one counseling toward greater emphasis on non-pharmacological approaches that integrate physiological and psychological regulatory mechanisms.^[13]^ HRV, being measurable, modifiable, and responsive to training, provides an objective foundation for such interventions, including breathing exercises, meditation, mindfulness practices, and physical activity. Exploring the interplay among resilience, vagal tone, and social adaptation thus not only advances theoretical understanding of left-behind children’s psychological development but also informs the design of practical, physiologically grounded interventions.

Against this background, the present study focuses on left-behind junior high school students in rural Hebei Province and employs a cross-sectional survey design to examine the relationships among psychological resilience, HRV (as an index of vagal tone), and social adaptation through both questionnaire and physiological measures. The objectives are to: test whether psychological resilience is associated with social adaptation in left-behind children; analyze whether vagal tone mediates this association; and provide theoretical and empirical support for HRV-based non-pharmacological interventions. This study broadens the physiological perspective in research on left-behind children’s psychology and offers reference points for innovation in mental health promotion and intervention models for children and adolescents. Specifically, we hypothesize that higher resilience will be associated with enhanced vagal tone, reflected in higher HRV, which in turn contributes to better social adaptation. This framework highlights HRV as a potential physiological mechanism linking resilience and adaptation, suggesting a partial mediation effect rather than a direct causal pathway.

## 2. Materials and methods

### 2.1. Research design

This research was approved by the Ethics Committee of Harrison International Peace Hospital. All participants and their guardians were fully informed about the study procedures, potential risks, and benefits. Since the participants were minors, written informed consent was obtained from their parents or legal guardians, and verbal assent was also obtained from the students themselves before data collection. Participation was entirely voluntary, and anonymity and confidentiality were strictly maintained. This study used cluster sampling to select first- and second-year students from 2 nonresidential middle schools located in the urban-rural fringe of Zaoqiang County. Ultimately, valid data were collected from 1481 participants, and after applying inclusion and exclusion criteria, a total of 312 participants were included in the final analysis.

The study utilized a cross-sectional design, aiming to explore the relationship among psychological resilience, vagal tone, and social adaptation ability. This design enables the collection of data at a specific point in time, facilitating the analysis of correlations between different variables. By assessing psychological resilience, vagal tone (HRV), and social adaptation ability of left-behind children, the study investigates the interrelationships among these 3 factors. The main objectives are:

To explore how psychological resilience and vagal tone influence social adaptation in left-behind children;
To verify the mediating role of vagal tone in the relationship between psychological resilience and social adaptation;
To provide a theoretical basis for non-pharmacological treatments and lay a foundation for future intervention strategies.

Definition of research subjects:

Left-behind children: refers to children whose parents have been working away from home for long periods and are cared for by other family members (such as grandparents or relatives).

### 2.2. Grouping and inclusion/exclusion criteria

High-resilience group: psychological resilience scale scores at or above the total sample mean + 0.5 standard deviations (M + 0.5SD); left-behind children; social adaptation scale scores above the mean + 0.5SD.

Low resilience group: psychological resilience scale scores at or below the total sample mean − 0.5 standard deviations (M − 0.5SD); left-behind children; social adaptation scale scores below the mean + 0.5SD.

This grouping criterion (M ± 0.5SD) has been adopted in previous psychological and educational studies to create clearly differentiated subgroups for comparison. It allows us to better examine the potential differences in HRV and social adaptation between adolescents with relatively high and low resilience levels.^[14]^

#### 2.2.1. Inclusion criteria

Left-behind children are defined as those whose one or both parents have been migrant workers for a cumulative period of more than five years, with less than one month of face-to-face contact with their parents in the past year, and who are cared for by grandparents or relatives, with identity confirmed by homeroom teachers or guardians. Participants must be in-school junior high students aged 11 to 15, with basic cognitive understanding and language expression ability, capable of independently completing questionnaires and experimental tasks; physically healthy, without major physiological or neurological diseases, and with no diagnosed history of mental disorders; behaviorally well-adjusted at school and cooperative in completing HRV monitoring and other testing procedures. Participants and their guardians must voluntarily sign the informed consent form.

#### 2.2.2. Exclusion criteria

Non–left-behind children were not included in the study; those already diagnosed with severe mental disorders such as major depression, generalized anxiety, schizophrenia, or autism were excluded; children with severe cardiovascular diseases, epilepsy, asthma, or other physical illnesses unsuitable for HRV measurement, or those currently taking medications affecting HRV, were also excluded; participants with severely missing or invalid data in measures of psychological resilience, social adaptation, or HRV, or those who were clearly uncooperative during the experiment, unwilling to participate, or whose parents did not sign the consent form, were not included in the analysis.

### 2.3. Data collection

Data collection in this study was divided into 3 main modules, corresponding to the 3 core variables: psychological resilience, vagal tone (HRV), and social adaptation, supplemented by demographic and background information to ensure a comprehensive assessment of the participants.

#### 2.3.1. Psychological resilience measurement

Psychological resilience was assessed using the Chinese version of the “Adolescent Psychological Resilience Scale (CD-RISC).” This scale, translated and revised by Chinese scholar Li Hailei, contains 40 items across 10 dimensions, including 6 external protective factors: peer support, family support, school support, school involvement, family involvement, and community support; and 4 internal trait factors: problem-solving, self-efficacy, empathy, and self-awareness.

#### 2.3.2. Social adaptation assessment

Social adaptation ability was measured using a localized and revised version of the “Social Adaptation Scale for Middle School Students.” This scale includes 5 dimensions: academic adaptation, interpersonal relationships, behavioral norms, emotional regulation, and sense of school belonging. It consists of 27 items rated on a 5-point Likert scale (1 = “strongly disagree” to 5 = “strongly agree”). The total score ranges from 27 to 135, with higher scores indicating stronger social adaptation ability. The internal consistency reliability of the scale is 0.91, indicating good structural validity.

#### 2.3.3. Vagal tone (HRV) assessment

HRV data were collected by trained professionals using portable HRV monitoring devices (e.g., Polar H10 and Kubios HRV Premium analysis software). Tests were conducted in quiet, well-ventilated, and disturbance-free classrooms. Participants were instructed to sit quietly for 5 minutes during the baseline HRV recording. Data collection took place between 16:00 and 17:30, avoiding measurements immediately after eating or vigorous physical activity. Key HRV indicators collected included:

High-frequency power (HF, unit: ms², reflecting parasympathetic nervous activity)
pNN50 (percentage of successive RR intervals differing by more than 50 ms)

The collected data were imported into KUBIOS HRV software for standardized analysis, with abnormal beats removed and both frequency-domain and time-domain parameters calculated.

#### 2.3.4. Demographic and background information

A structured questionnaire was used to collect demographic and life background variables, including age, gender, physical health status, parental migration history, guardian identity, family economic status, academic performance, physical activity, sleep quality (PSQI), and anxiety level (GAD-7 scale), with some information verified by class teachers or guardians. The questionnaire was distributed and completed in class under the guidance of trained researchers, with an average completion time of approximately 30 minutes.

### 2.4. Statistical analysis

Statistical analyses were performed using SPSS 26.0 and AMOS 24.0. Descriptive statistics were first conducted to present the basic characteristics of the sample. Independent samples *t*-tests, chi-square tests, and non-parametric tests were used to compare differences between high and low resilience groups across variables. After controlling for confounding factors such as age, gender, sleep quality, and anxiety, ANCOVA (analysis of covariance) was used to test the effects of psychological resilience on HRV indicators and social adaptation ability. Pearson correlation analysis was used to explore correlations among psychological resilience, HRV, and social adaptation, and Bootstrap mediation analysis was employed to test the mediating role of HRV between psychological resilience and social adaptation. In addition, univariate logistic regression was used to screen significant variables related to social adaptation, followed by the construction of a multiple linear regression model to further identify the independent predictive roles of psychological resilience and HRV on social adaptation. A two-tailed *P*-value of <.05 was considered statistically significant.

## 3. Results

### 3.1. Basic information and differences between high and low resilience groups

A total of 312 left-behind children were included in this study, with 156 children in the high-resilience group and 156 children in the low resilience group. The average age of the high-resilience group was 12.5 ± 1.2 years, which was lower than that of the low resilience group at 13.0 ± 1.3 years. The proportion of boys was 59.6% in the high-resilience group and 46.2% in the low resilience group. The difference in resilience scores between the two groups was significant (28.5 ± 3.1 vs. 15.2 ± 2.8, *P* <.001), validating the effectiveness of the grouping. In addition, significant differences were found between the two groups in terms of years of parental migration, frequency of communication with parents, family economic status, frequency of family conflicts, academic adjustment, health behaviors, social support, and other dimensions, as shown in Table 1.

**Table 1:** Group differences in baseline characteristics of high and low resilience left-behind children.

Variable category	Specific indicator	High-resilience group (n = 156)	Low resilience group (n = 156)	Test statistic	*P*-value
Grouping variable	Resilience score (CD-RISC)	28.5 ± 3.1	15.2 ± 2.8	t = 105.33	<.001
Demographics	Age (year)	12.5 ± 1.2	13.0 ± 1.3	t = 6.27	<.001
	Male	93 (59.6%)	72 (46.2%)	χ² = 5.84	.016
Parental migration	Years of parental migration	4.8 (3.0, 6.0)	6.5 (4.0, 9.0)	Z = –8.97	<.001
	Call frequency with parents (≥ once/week)	108 (69.2%)	50 (32.1%)	χ² = 104.75	<.001
	Both parents migrant	112 (71.8%)	65 (41.6%)	χ² = 3.12	.077
Family environment	Primary caregiver: grandparents	133 (85.3%)	67 (43.4%)	χ² = 5.66	.017
	Monthly family income < ¥3000	59 (37.8%)	82 (52.6%)	χ² = 95.32	<.001
	Family conflict frequency (≥ once/week)	19 (12.2%)	43 (27.6%)	χ² = 90.34	<.001
	Guardian education ≥ junior high school	67 (42.9%)	34 (21.8%)	χ² = 45.21	<.001
School adjustment	Top 30% in academic rank	88 (56.4%)	56 (35.9%)	χ² = 120.56	<.001
	Monthly teacher counseling (≥ once/month)	84 (53.8%)	60 (38.5%)	χ² = 135.80	<.001
Physical health	BMI (kg/m²)	18.2 (17.0, 19.6)	17.7 (16.5, 19.1)	Z = –4.02	<.001
	History of chronic disease	8 (5.1%)	25 (16%)	χ² = 6.45	.011
	Poor sleep quality (PSQI ≥ 8)	32 (20.5%)	65 (41.7%)	χ² = 160.22	<.001
Psychological/behavioral	Anxiety symptoms (GAD-7 ≥ 5)	39 (25%)	112 (72%)	χ² = 200.15	<.001
	Daily screen time (hours)	1.4 (1.0, 2.0)	3.1 (2.2, 4.0)	Z = –27.63	<.001
	Physical exercise (≥ 3 times/week)	101 (64.8%)	36 (23.1%)	χ² = 171.20	<.001
Social support	Perceived teacher support (high)	122 (78.2%)	97 (62%)	χ² = 222.15	<.001
	Peer relationship satisfaction (high)	109 (69.9%)	75 (48%)	χ² = 187.64	<.001
Negative experiences	Bullying experience (yes)	18 (12.0%)	132 (80%)	χ² = 73.18	<.001

BMI = body mass index, CD-RISC = Connor–Davidson resilience scale, GAD-7 = generalized anxiety disorder-7 scale, PSQI = Pittsburgh sleep quality index.

### 3.2. Effects of psychological resilience on HRV and social adaptation: ANCOVA results

After controlling for covariates such as age, gender, family conflict, sleep quality, and anxiety, significant differences in HRV and social adaptation remained between the high and low resilience groups (see Table 2). The high-resilience group had a mean HRV high-frequency power of 690.4 ± 86.3 ms², significantly higher than the low resilience group’s 645.7 ± 79.2 ms² (F = 12.74, *P* = .0004, partial η² = 0.036), indicating a moderate effect size. HRV-pNN50 was also higher in the high-resilience group (38.1% ± 6.7 vs. 34.3% ± 6.1, F = 21.31, *P* <.001, partial η² = 0.058), again reflecting a moderate effect size. The difference in social adaptation was the most significant, with the high-resilience group scoring 108.2 ± 8.4 and the low resilience group scoring 100.1 ± 9.0 (F = 54.89, *P* <.001, partial η² = 0.162), indicating a large effect size. These findings demonstrate that psychological resilience has a significant impact on social adaptation.

**Table 2:** ANCOVA results comparing high and low resilience groups.

Variable	High-resilience group (mean ± SD)	Low resilience group (mean ± SD)	*F*-value	*P*-value	Partial η²	Effect size
HRV high-frequency power (ms²)	690.4 ± 86.3	645.7 ± 79.2	12.74	.0004	0.036	Medium
HRV-pNN50 (%)	38.1 ± 6.7	34.3 ± 6.1	21.31	<.001	0.058	Medium
Social adaptation score	108.2 ± 8.4	100.1 ± 9.0	54.89	<.001	0.162	**Large**

ANCOVA = analysis of covariance, HRV = heart rate variability, pNN50 = percentage of successive RR intervals differing by more than 50 ms, SD = standard deviation.

### 3.3. Correlation analysis of psychological resilience, HRV, and social adaptation

The results of Pearson correlation analysis showed that psychological resilience was highly positively correlated with social adaptation (*R* = 0.95, *P* <.01), and also significantly correlated with both HRV indicators: HRV-HF (*R* = 0.59, *P* <.01) and HRV-pNN50 (*R* = 0.81, *P* <.01), as shown in Table 3. In addition, HRV-HF and HRV-pNN50 were correlated with each other (*R* = 0.51), and both were moderately to strongly positively correlated with social adaptation (*R* = 0.56 and *R* = 0.77, respectively, both *P* <.01). These results support the existence of close associations among the three variables.

**Table 3:** Correlation matrix of resilience, HRV, and social adaptation.

	Resilience	HRV-HF	HRV–pNN50	Social adaptation
Resilience	1	0.59**	0.81**	0.95**
HRV-HF	0.59**	1	0.51**	0.56**
HRV–pNN50	0.81**	0.51**	1	0.77**
Social adaptation	0.95**	0.56**	0.77**	1

Pearson correlation coefficients are reported.

HF = high-frequency power (component of HRV), HRV = heart rate variability, pNN50 = percentage of successive RR intervals differing by more than 50 ms.

* *P* <.05.

** *P* <.01.

### 3.4. Mediating role of HRV between psychological resilience and social adaptation

The mediation analysis results indicated that both HRV indicators (high-frequency power and pNN50) played significant mediating roles in the pathway from psychological resilience to social adaptation. Specifically, psychological resilience significantly positively predicted HRV high-frequency power (path a: B = 7.85, *P* = .001), and HRV high-frequency power further significantly predicted social adaptation levels (path b: B = 0.032, *P* = .004). The indirect effect was 0.25 (95% CI [0.09, 0.46], *P* = .006), which, although statistically significant, represents a relatively small effect size, indicating that HRV-HF functioned only as a partial mediator in the relationship between psychological resilience and social adaptation (see Table 4).

**Table 4:** Mediation analysis of HRV-HF between resilience and social adaptation.

Pathway	Coefficient	Boot SE	95% CI	*P*-value
Resilience → HRV-HF (a path)	7.85	1.23	(5.42–10.02)	.001
HRV-HF → social adaptation (b path)	0.032	0.011	(0.010–0.054)	.004
Direct effect (resilience → adaptation)	2.86	0.34	(2.20–3.48)	<.001
Indirect effect (a × b)	0.25	0.09	(0.09–0.46)	.006

CI = confidence interval, HF = high-frequency power (component of HRV), HRV = heart rate variability.

Similarly, psychological resilience significantly predicted HRV-pNN50 (B = 1.78, *P* <.001), and HRV-pNN50 had a positive predictive effect on social adaptation (B = 0.14, *P* = .003). The indirect effect was 0.25 (95% CI [0.11, 0.43], *P* = .007), further supporting its role as a significant mediating variable (see Table 5).

**Table 5:** Mediation analysis of HRV-pNN50 as a mediator between resilience and social adaptation.

Pathway	Coefficient	Boot SE	95% CI	*P*-value
Resilience → HRV-pNN50 (a path)	1.78	0.32	(1.16–2.41)	<.001
HRV-pNN50 → social adaptation (b path)	0.14	0.05	(0.05–0.23)	.003
Direct effect (resilience → adaptation)	2.65	0.33	(2.01–3.28)	<.001
Indirect effect (a × b)	0.25	0.08	(0.11–0.43)	.007

CI = confidence interval, HRV = heart rate variability, pNN50 = percentage of successive RR intervals differing by more than 50 ms.

### 3.5. Univariate logistic regression analysis: potential factors influencing social adaptation ability

The results of univariate logistic regression analysis showed that psychological resilience (OR = 1.20, 95% CI: 1.15–1.26, *P* <.001) and HRV indicators (HRV-HF: OR = 1.01, *P* = .003; HRV-pNN50: OR = 1.04, *P* <.001) were both significant positive predictors of social adaptation ability. Younger age (OR = 0.81, *P* = .001), female gender (OR = 0.60, *P* = .003), fewer family conflicts (OR = 0.47, *P* <.001), better sleep quality (OR = 0.42, *P* = .001), and lower anxiety levels (OR = 0.40, *P* = .001) were also significantly associated with higher levels of social adaptation.

In contrast, variables such as BMI, chronic illness, screen time, physical activity, teacher support, peer relationship satisfaction, and school bullying did not reach statistical significance in this model (*P* >.05), as detailed in Table 6.

**Table 6:** Univariate logistic regression predicting high social adaptation.

Predictor	B	SE	Wald	OR (95% CI)	*P*-value
Resilience	0.185	0.024	59.418	1.20 (1.15–1.26)	<.001
HRV-HF	0.006	0.002	9	1.01 (1.00–1.01)	.003
HRV-pNN50	0.042	0.011	14.579	1.04 (1.02–1.07)	<.001
Age	−0.208	0.063	10.9	0.81 (0.72–0.92)	.001
Gender (male = 1)	−0.516	0.174	8.794	0.60 (0.42–0.84)	.003
Family conflict (≥1/wk)	−0.745	0.194	14.793	0.47 (0.32–0.67)	<.001
Sleep quality (PSQI ≥ 8)	−0.863	0.251	11.799	0.42 (0.25–0.70)	.001
Anxiety symptoms (GAD-7 ≥ 5)	−0.928	0.278	11.125	0.40 (0.23–0.69)	.001
BMI	0.015	0.054	0.078	1.02 (0.91–1.14)	.78
Chronic disease	−0.233	0.192	1.473	0.79 (0.55–1.13)	.225
Screen time	−0.059	0.039	2.294	0.94 (0.87–1.01)	.13
Physical exercise	0.121	0.128	0.894	1.13 (0.88–1.44)	.344
Teacher support	0.153	0.157	0.949	1.17 (0.86–1.59)	.33
Peer satisfaction	0.102	0.141	0.518	1.11 (0.84–1.47)	.472
Bullying	−0.285	0.183	2.426	0.75 (0.53–1.06)	.119

BMI = body mass index, CI = confidence interval, GAD-7 = generalized anxiety disorder-7 scale, HF = high-frequency power (component of HRV), HRV = heart rate variability, OR = odds ratio, pNN50 = percentage of successive RR intervals differing by more than 50 ms, PSQI = Pittsburgh sleep quality index.

### 3.6. Multiple linear regression analysis of psychological resilience and HRV indicators on social adaptation

To further clarify the independent predictive effects of psychological resilience and vagal tone on social adaptation, this study constructed a multiple linear regression model using social adaptation scores as the dependent variable. Psychological resilience, HRV-HF, and HRV-pNN50 were included as main independent variables, while controlling for confounding factors such as age, gender, family conflict, sleep quality, and anxiety symptoms.

The results showed that psychological resilience had a significant positive predictive effect on social adaptation (B = 2.12, *P* <.001), and HRV-pNN50 also showed a significant positive prediction (B = 0.14, *P* = .005). However, HRV-HF was no longer significant after controlling for other variables (B = 0.015, *P* = .095). Among the control variables, age, male gender, weekly family conflict, poor sleep quality (PSQI ≥ 8), and presence of anxiety symptoms (GAD-7 ≥ 5) were all significantly negatively associated with social adaptation (all *P* <.05). See Table 7 for details.

**Table 7:** Multiple linear regression predicting social adaptation.

Predictor	B	SE	β	*t*	*P*
Resilience	2.12	0.21	0.61	10.1	<.001
HRV-HF	0.015	0.009	0.17	1.67	.095
HRV-pNN50	0.14	0.05	0.22	2.8	.005
Age	−0.26	0.12	−0.09	-2.17	.03
Gender (male = 1)	−1.15	0.48	−0.12	-2.4	.017
Family conflict (≥1/wk)	−2.01	0.62	−0.2	−3.24	.001
Sleep quality (PSQI ≥ 8)	−1.03	0.3	−0.18	−3.43	.001
Anxiety symptoms (GAD-7 ≥5)	−1.89	0.55	−0.23	−3.44	.001

GAD-7 = generalized anxiety disorder-7 scale, HF = high-frequency power (component of HRV), HRV = heart rate variability, pNN50 = percentage of successive RR intervals differing by more than 50 ms, PSQI = Pittsburgh sleep quality index.

## 4. Discussion

This study systematically examined the psychological–physiological–behavioral mechanisms linking resilience, vagal tone (HRV), and social adaptation in left-behind children. Based on survey data and physiological measurements from 312 middle school students, we found that resilience significantly predicted social adaptation and showed moderate to strong correlations with HRV indicators (HF and pNN50). Mediation analyses further indicated that HRV partially mediated the relationship between resilience and social adaptation, underscoring the role of internal regulatory mechanisms in shaping external behavioral outcomes. These findings suggest that resilience is closely associated with social adaptation in left-behind children and that vagal tone may serve as a key physiological pathway underlying this link.

First, children with higher psychological resilience tended to exhibit better social adaptation compared to those with lower resilience. This finding aligns with previous studies in general adolescent populations. As a cognitive and behavioral resource for coping with stress, frustration, and adversity, resilience plays a strong protective role.^[14]^ For left-behind children, who often experience prolonged parental separation and limited emotional support, higher resilience may facilitate the regulation of negative emotions, maintenance of interpersonal relationships, and preservation of learning motivation and self-esteem, thereby improving overall adaptation in school and social contexts.^[15]^ This highlights the urgency and practical importance of fostering resilience among this vulnerable group.

Second, resilience was not only strongly related to social adaptation but also highly correlated with HRV, particularly pNN50, where the correlation reached 0.81. This finding supports a physiological basis for resilience through autonomic regulation. From a neurophysiological perspective, HRV reflects vagal function and the flexibility of self-regulation systems. Higher HRV indicates stronger capacity to recover from arousal states and to modulate emotional and cognitive responses.^[16]^ Thus, resilience can be regarded as the psychological manifestation of such physiological flexibility, with higher resilience corresponding to more efficient parasympathetic regulation.

ANCOVA results further confirmed that, even after controlling for factors such as age, gender, sleep quality, and anxiety, the high-resilience group displayed significant advantages in both HRV (HF and pNN50) and social adaptation. This indicates that the beneficial effects of resilience on vagal tone and adaptation are not merely attributable to background or health-related factors but instead operate through a relatively independent psychological–physiological pathway.^[17,18]^ Mediation analysis additionally revealed that HRV partially mediated the association between resilience and adaptation. However, the indirect effect was relatively small (0.25), suggesting that vagal tone is not the sole pathway. Other mechanisms – such as emotional regulation strategies, coping styles, and social support – may also play important mediating or moderating roles. Future research should examine these additional pathways to establish a more comprehensive explanatory model.

Regression analyses showed that resilience and HRV-pNN50 remained significant predictors of social adaptation even after controlling for confounders, while HRV-HF lost significance in the multivariate model. This suggests that pNN50 may be a more sensitive indicator than HF in adolescents. Time-domain parameters like pNN50 may better capture actual vagal regulation during this developmental stage because adolescents’ autonomic nervous systems are still maturing, and their variable breathing patterns reduce the stability of frequency-domain measures such as HF. In contrast, adult studies frequently report HF as a robust vagal marker, whereas adolescent studies highlight stronger associations for time-domain indices such as RMSSD or pNN50. These developmental differences may explain why pNN50 emerged as a stronger predictor in our sample, offering clearer measurement guidance for future non-pharmacological intervention research.^[19]^

Several limitations should be noted. First, the cross-sectional design precludes causal inference; longitudinal studies are needed to clarify temporal and dynamic relationships among resilience, HRV, and adaptation. Second, HRV was measured only during short-term resting-state recordings, which do not capture regulatory responses under stress. Third, while we controlled for some confounders (e.g., physical constitution, diet, sleep), other factors remain unmeasured. In particular, family structure, parenting style, and the quality of parent–child relationships may also influence resilience, HRV, and social adaptation and should be included in future studies. Fourth, although the sample size was adequate, participants were drawn from only 2 schools in 1 region of Hebei Province, limiting generalizability across China’s diverse cultural and socioeconomic contexts. Finally, this study did not include an intervention component. Future work should incorporate experimental designs testing HRV-based approaches such as breathing training, mindfulness, or exercise to assess their practical effectiveness in improving resilience and adaptation.

In practical terms, our findings suggest that HRV-informed interventions could be feasibly integrated into school and rural settings. For instance, breathing exercises or mindfulness sessions could be embedded in school health curricula with minimal resource requirements, while group activities and simple HRV biofeedback tools may provide low-cost options in rural schools lacking professional psychological services. Training teachers and school counselors in these techniques could help build sustainable support systems. Such strategies align with the physiological evidence of this study and offer realistic, scalable approaches to promote the mental health of left-behind children in resource-limited contexts.

In conclusion, this study empirically validated the close associations among resilience, vagal tone, and social adaptation and provided the first evidence that HRV partially mediates the resilience–adaptation link in left-behind children. These findings enrich theoretical models of social adaptation and provide a foundation for developing HRV-based non-pharmacological interventions. Future research should expand sample diversity, adopt longitudinal and experimental designs, and promote applied interventions to support the mental health of high-risk youth groups such as left-behind children.

## Author contributions

**Conceptualization:** Xuejing Liu, Longhuan Zhang.

**Data curation:** Xuejing Liu, Longhuan Zhang, Meiying Zhang.

**Formal analysis:** Xuejing Liu, Longhuan Zhang, Meiying Zhang, Ningning Li.

**Investigation:** Longhuan Zhang.

**Methodology:** Xuejing Liu.

**Project administration:** Ningning Li.

**Resources:** Ningning Li.

**Software:** Ningning Li.

**Supervision:** Xuejing Liu, Longhuan Zhang, Meiying Zhang, Ningning Li.

**Validation:** Xuejing Liu, Longhuan Zhang, Meiying Zhang, Ningning Li.

**Visualization:** Xuejing Liu, Longhuan Zhang, Meiying Zhang, Ningning Li.

**Writing – original draft:** Xuejing Liu.

**Writing – review & editing:** Xuejing Liu.
